# Perspectives of healthcare professionals in Qatar on causes of medication errors: A mixed methods study of safety culture

**DOI:** 10.1371/journal.pone.0204801

**Published:** 2018-09-28

**Authors:** Derek Stewart, Binny Thomas, Katie MacLure, Abdulrouf Pallivalapila, Wessam El Kassem, Ahmed Awaisu, James S. McLay, Kerry Wilbur, Kyle Wilby, Cristin Ryan, Andrea Dijkstra, Rajvir Singh, Moza Al Hail

**Affiliations:** 1 School of Pharmacy and Life Sciences, Robert Gordon University, Aberdeen, United Kingdom; 2 Women’s Hospital, Hamad Medical Corporation, Doha, Qatar; 3 College of Pharmacy, Qatar University, Doha, Qatar; 4 Institute of Medical Sciences, University of Aberdeen, Aberdeen, United Kingdom; 5 School of Pharmacy, Royal College of Surgeons in Ireland, Dublin, Ireland, Dublin 2; 6 Hamad Medical Corporation, Doha, Qatar; Newcastle University, UNITED KINGDOM

## Abstract

**Background:**

There is a lack of robust, rigorous mixed methods studies of patient safety culture generally and notably those which incorporate behavioural theories of change. The study aimed to quantify and explain key aspects of patient safety culture which were of most concern to healthcare professionals in Qatar.

**Methods:**

A sequential explanatory mixed methods design of a cross-sectional survey followed by focus groups in Hamad Medical Corporation, Qatar. All doctors, nurses and pharmacists were invited to complete the Hospital Survey on Patient Safety Culture (HSOPS). Respondents expressing interest in focus group participation were sampled purposively, and discussions based on survey findings using the Theoretical Domains Framework (TDF) to explain behavioural determinants.

**Results:**

One thousand, six hundred and four questionnaires were received (67.9% nurses, 13.3% doctors, 12.9% pharmacists). HSOPS composites with the lowest levels of positive responses were non-punitive response to errors (24.0% positive) and staffing (36.2%). Specific TDF determinants potentially associated with these composites were social/professional role and identity, emotions, and environmental context and resources. Thematic analysis identified issues of doctors relying on pharmacists to correct their errors and being reluctant to alter the prescribing of fellow doctors. There was a lack of recognition of nurses’ roles and frequent policy non-adherence. Stress, workload and lack of staff at key times were perceived to be major contributors to errors.

**Conclusions:**

This study has quantified areas of concern relating to patient safety culture in Qatar and suggested important behavioural determinants. Rather than focusing on changing behaviour at the individual practitioner level, action may be required at the organisational strategic level to review policies, structures (including resource allocation and distribution) and processes which aim to promote patient safety culture.

## Introduction

Promoting patient safety in healthcare settings is a global challenge, with an estimated one in ten patients being harmed whilst receiving care [1]. In an effort to raise awareness of key concepts and strategies in patient safety, the World Health Organization (WHO) published ‘Medication Without Harm, WHO Global Patient Safety Challenge’ in March 2017 [2,3]. The challenge calls for action to reduce patient harm which occurs as a result of unsafe medication practices and medication errors [2,3]. The goal is to ‘gain worldwide commitment and action to reduce severe, avoidable medication-related harm by 50% in the next five years, specifically by addressing harm resulting from errors or unsafe practices due to weaknesses in health systems’. Accumulation of evidence confirms that healthcare professionals often prescribe, dispense and administer medication in ways and circumstances that may increase the risk of patient harm [4–8].

Whilst it is noted that the magnitude and nature of medication harm may differ between countries, globally the cost associated with medication errors has been estimated at US$ 42 billion annually [2,3]. The most commonly cited and accepted definition of the term ‘medication error’ is that of the National Coordinating Council for Medication Error Reporting and Prevention (NCCMERP) in the United States (US), ‘any preventable event that may cause or lead to inappropriate medication use or patient harm, while the medication is in the control of the health care professional, patient, or consumer’ [9]. Most research literature focuses on errors relating to prescribing, administration and dispensing, with evidence that causation is often complex and multifactorial. Systematic reviews have focused on causes of medication errors in different patient populations and settings [10–13]. Common to all systematic reviews is the relatively poor research methodologies reported in most of the primary literature, a lack of behavioural theory and organisational culture in study design. Furthermore, very few studies employed a mixed methods approach to allow quantification and in-depth description and explanation of contributory factors.

Behavioural theories may be used to provide explanation hence providing a robust and rigorous foundation for development of behaviour change interventions. The United Kingdom (UK) Medical Research Council (MRC) framework, ‘Developing and implementing complex interventions’ highlights the importance of considering theory, noting that interventions grounded in theory are more likely to be effective than those developed empirically or pragmatically’ [14]. The Theoretical Domains Framework (TDF) is being used increasingly within health-related research to provide insight into influences on behaviour. TDF derives from 33 psychological theories and 128 theoretical constructs organised into 14 domains of behavioural determinants, as described in Table 1 [15].

**Table 1:** Description of TDF domains (adapted from Cain et al. [15]).

TDF Domains	Description
Knowledge	An awareness of the existence of something
Skills	An ability or proficiency acquired through practice
Social/Professional Role and Identity	A coherent set of behaviours and displayed personal qualities of an individual in a social or work setting
Beliefs about Capabilities	Acceptance of the truth, reality, or validity about an ability, talent, or facility that a person can put to constructive use
Optimism	The confidence that things will happen for the best or that desired goals will be attained
Beliefs about Consequences	Acceptance of the truth, reality, or validity about outcomes of a behaviour in a given situation
Reinforcement	Increasing the probability of a response by arranging a dependent relationship, or contingency, between the response and a given stimulus
Intentions	A conscious decision to perform a behaviour or a resolve to act in a certain way
Goals	Mental representations of outcomes or end states that an individual wants to achieve
Memory, Attention and Decision Processes	The ability to retain information, focus selectively on aspects of the environment and choose between two or more alternatives
Environmental Context and Resources	Any circumstance of a person's situation or environment that discourages or encourages the development of skills and abilities, independence, social competence, and adaptive behaviour
Social Influences	Those interpersonal processes that can cause individuals to change their thoughts, feelings, or behaviours
Emotion	A complex reaction pattern, involving experiential, behavioural, and physiological elements, by which the individual attempts to deal with a personally significant matter or event
Behavioural Regulation or measured actions	Anything aimed at managing or changing objectively observed

It is apparent that there is also a need to focus attention on organisational safety culture. The ‘Study Group on Human Factors’ defines organisational safety culture as, ‘the product of individual and group values, attitudes, perceptions, competencies, and patterns of behaviour that determine the commitment to, and the style and proficiency of, an organization’s health and safety management [16].’ While two systematic reviews have explored interventions to promote safety culture in hospitals in general and acute hospitals specifically, medication safety was not a feature of any primary research [17,18].

In an attempt to promote and standardise the measurement of organisational safety culture, the US Agency for Healthcare Research and Quality (AHRQ) and Medical Errors Workgroup of the Quality Interagency Coordination Task Force (QuIC) sponsored the development of the Hospital Survey on Patient Safety Culture (HSOPS) [19]. Items are clustered within 12 composites as presented in Table 2.

**Table 2:** HSOPS composites and definitions [19].

Composite	Definition: The extent to which…
Communication openness	staff freely speak up if they see something that may negatively affect a patient and feel free to question those with more authority
Feedback and communication about error	staff are informed about errors that happen, are given feedback about changes implemented, and discuss ways to prevent errors
Frequency of events reported	mistakes of the following types are reported: mistakes caught and corrected before affecting the patient; mistakes with no potential to harm the patient; and mistakes that could harm the patient but do not
Handoffs and transitions	important patient care information is transferred across hospital units and during shift changes
Management support for patient safety	hospital management provides a work climate that promotes patient safety and shows that patient safety top priority
Non-punitive response to error	staff feel that their mistakes and event reports are not held against them and that mistakes are not kept in their personnel file
Organisational learning—continuous improvement	mistakes have led to positive changes and changes evaluated for effectiveness
Overall perceptions of patient safety	procedures and systems are good at preventing errors and there is a lack of patient safety problems
Staffing	there are enough staff to handle the workload and work hours are appropriate to provide the best care for patients
Supervisor/manager expectations and actions promoting patient safety	supervisors/managers consider staff suggestions for improving patient safety, praise staff for following patient safety procedures, and do not overlook patient safety problems
Teamwork across units	hospital units cooperate and coordinate with one another to provide the best care for patients
Teamwork within units	staff support each other, treat each other with respect, and work together as a team

Research on medication errors within the Middle East has historically been reported to be of poor quality [20]. Recently, Elmontsri et al. conducted a systematic review to explore the status of patient safety culture in Arab countries based on the findings of the HSOPS [21]. Data from 18 studies across seven countries (excluding Qatar) were included, identifying that composites relating to non-punitive response to error to be infrequently practised in their organisation, that staffing levels were often inadequate and that communication needed to be more open. The authors concluded that further research is warranted to provide explanation of these findings and to identify potential interventions to enhance culture and patient safety.

The aim of the present study was to quantify and explain key aspects of patient safety culture which were of most concern to health professionals in Qatar.

## Methods

### Study design

A sequential explanatory mixed methods design was employed, with a cross-sectional survey followed by focus groups in samples of questionnaire respondents to provide further depth and explanation of survey findings [22,23].

### Setting

The research was conducted within Hamad Medical Corporation (HMC), the main provider of secondary and tertiary healthcare in Qatar.

### Cross-sectional survey

The first phase of the research was a cross-sectional survey.

### Questionnaire development

The questionnaire was adapted from AHRQ HSOPS with items presented as 5-point Likert type scales; personal and practice demographic items were added. The common language of care delivery at HMC is English thus translation into other languages (e.g. Arabic) was not required. The questionnaire was piloted in a convenience sample of 100 healthcare professionals. Test-retest reliability was assessed in pilot respondents by requesting that the questionnaire be completed on a second occasion within an interval of two weeks. A high level of test-retest reliability was achieved (p<0.001, Cohen’s kappa) for all Likert statements. The final questionnaire was formatted in Snap 10 Professional (software for web and email questionnaire design, publication, data entry and analysis). On completion of the questionnaire, respondents were invited to participate in focus groups to discuss responses in more detail.

### Recruitment

All doctors, nurses and pharmacists working within HMC were eligible to participate, with no exclusions. Three hundred and sixty responses were required to give a margin of error of 5% with 95% confidence intervals [24]. Online participation was encouraged via HMC web alerts and promotional posters; paper-based questionnaires were distributed to all doctors, nurses and pharmacists. Data were collected from mid-January 2016 to mid-April 2016.

### Data analysis

Anonymised online submissions were imported into Snap before direct export to SPSS version 21.0 and data cleaned prior to analysis. Paper-based questionnaire data were entered manually using the survey link. Descriptive statistics were used to describe respondents’ demographic and professional characteristics as well as their responses to individual HSOPS safety culture items. For each composite, a score was determined in line with the recommendations of the AHRQ task force [19]. This involved firstly calculating the percentage of positive responses to each item. A positive response may reflect agreement with a positively phrased statement, or may reflect disagreement with a negatively phrased statement. Therefore, for positively worded items the percentage of positive responses is the proportion of ‘agree’ and ‘strongly agree’ responses to the item, and for negatively worded items the percentage of positive responses is the proportion of disagree and strongly disagree responses to the item. Higher percentages (%) therefore reflect more positive responses to HSOPS safety culture items as defined in Table 2. The composite score was then expressed as the mean of the positive responses. For those composites with overall positive responses of <50%, Chi-square was used to determine any statistically significant associations with demographic variables.

### Focus groups

To clarify, explore and explain issues identified in the survey phase, a qualitative approach was employed.

### Sampling and recruitment

Respondents of the survey who expressed interest in participating in the focus groups were sampled purposively to represent a range of professions, hospitals and number of years of experience.

### Data generation

The focus group topic guide was developed following analysis of the questionnaire data, with reference to the TDF [15]. Initial discussions were based around views and experiences on safety culture in relation to causes of medication errors. This was followed by targeted discussions around patient cases of errors illustrating errors in prescribing, dispensing and administration in diverse patient groups which led to significant harm. TDF domains were used as prompts in relation to potential causes of errors. Focus groups were moderated by two experienced qualitative researchers, with informed consent obtained from each participant at the outset. Discussions were audio-recorded (with permission), transcribed verbatim and checked for transcribing reliability. A clear audit trail was maintained which documented details of data gathering to promote dependability [25]. Sampling and recruitment continued to the point of data saturation, when no new themes emerged from data analysis [26]. Focus groups were conducted between mid-May 2016 and mid-June 2016.

### Data analysis

Data analysis followed the Framework Approach, using TDF domains deductively for to generate a coding framework [27]. Two researchers independently read each focus group transcript repeatedly to ensure familiarity, then coded text to one or more TDF domains. Any disagreements were resolved by discussion which involved a third researcher if needed.

### Ethics

The study received ethical approval from Hamad Medical Corporation, Medical Research Center Qatar, Qatar University Institutional Review Board and Robert Gordon University Research Ethics Sub-Committee. Return of the questionnaire was taken as an indication of informed consent; written informed consent was obtained from all focus group participants.

## Results

### Cross-sectional survey

#### Respondents’ demographics and professional characteristics

One thousand, six hundred and four completed questionnaires were received, with most (67.9%) from nurses followed by doctors (13.3%) and pharmacists (12.9%). Around three quarters (70.9%) were female, <40 years (76.0%) and almost half (48.1%) with more than 10 years of experience as healthcare professionals. Respondents had varying involvement with medicines-related processes as follows: prescribing medicines (15.1%); administering (61.1%); preparation and dispensing (25.9%); and monitoring (42.0%) (Table 3).

**Table 3:** Respondents’ demographic and professional characteristics (N = 1604).

Characteristic	Percentage	Frequency, n
***Current role in the hospital***		
Clinical nurse educator	0.7	12
Clinical pharmacist	2.8	45
Consultant physician	5.4	86
Head/Charge/Specialist nurse	17.1	275
Nurse	50.0	802
Pharmacist	8.9	143
Pharmacy Director/Supervisor/Specialist	1.2	19
Resident Physician	3.5	56
Specialist Physician	4.5	72
Other	5.0	80
Missing	0.9	14
***Age (years)***		
≤29	24.2	392
30–39	41.8	670
40–49	21.5	345
50–59	9.5	153
≥60	1.6	25
Missing	1.7	19
***Gender***		
Male	27.6	442
Female	70.9	1137
Missing	1.6	25
***Country of receiving entry-to-practice degree***		
India	42.7	685
Philippines	17.6	283
Egypt	9.3	149
Qatar	9.2	148
Jordan	4.8	77
Other	14.5	231
Missing	1.9	31
***Experience as healthcare professional in hospital (years)***		
<1	1.6	25
1–5	19.1	306
6–10	29.4	471
11–15	21.4	343
16–20	12.0	193
>20	14.7	235
Missing	1.9	31
***Experience as healthcare professional in Qatar (years)***		
<1	8.5	136
1–5	40.3	647
6–10	21.8	350
11–15	16.5	264
16–20	5.1	82
>20	6.7	108
Missing	1.1	17
***Hours worked in a typical week***		
<20	1.3	21
20–39	10.6	170
40–59	82.7	1326
≥60	3.0	48
Missing	2.4	39
***In your role you typically have direct interaction or contact with patients***		
Yes	85.6	1373
No	9.0	145
Missing	5.4	86
***Your primary roles in the medicines process are (multiple options could be chosen)***		
Prescribing	15.1	243
Administering	61.1	980
Preparation and Dispensing	25.9	415
Monitoring	42.0	673
Missing	3.1	49

#### Patient safety culture items

Positive responses to the HSOPS composites and items are given in Table 4. Composites with the lowest levels of mean positive responses were: non-punitive response to errors (24.0%); staffing (36.2%); communication openness (50.5%); handoffs and transitions (53.1%); and supervisor/manager expectations and actions promoting patient safety (56.5%). Composites with the highest levels of positive responses were: organisational learning–continuous improvement (85.5%); team working within unit (82.1%); and management support for patient safety (75.4%). For the two composites with mean positive responses of <50%, Chi-square was used to determine the associations between percentage positive responses and demographics/professional characteristics.

**Table 4:** Positive responses to HSOPS items and composites (N = 1604).

Statements	% positive response (100% representing the highest positive response to each statement)
***Non-punitive response to errors***, overall positive response = 24.0%**	
*Staff feel like errors count against them	26.2 (disagreed)
*When an error is reported, it feels like the person is being reported, not the problem	31.1 (disagreed)
*Staff worry that errors they make are kept in their personnel file	14.6 (disagreed)
***Staffing***, overall positive response = 36.2%	
We have enough staff to handle the workload	54.7 (agreed)
*We use more locum staff than is best for patient care	30.5 (disagreed)
*We work under pressure trying to do too much, too quickly	23.5 (disagreed)
***Communication openness***, overall positive response = 50.5%	
Staff will speak up freely if they see something that may negatively affect patient care	60.9 (agreed)
Staff feel free to question the decisions or actions of those with more authority	46.6 (agreed)
*In this unit, staff are afraid to ask questions when something does not seem right	44.0 (disagreed)
***Handoffs and transitions***, overall positive response = 53.1%	
*Things get missed when transferring patients from one unit to another	53.7 (disagreed)
*Important patient care information is often lost during shift changes	60.8 (disagreed)
*Problems often occur in the exchange of information across hospital units	42.9 (disagreed)
*Shift changes are problematic for patients in this hospital	55.1 (disagreed)
***Supervisor/manager expectations and actions promoting patient safety***, overall positive response = 56.5%	
My supervisor/ manager says a good word when he/she sees a job done according to established patient safety procedures	73.0 (agreed)
My supervisor/ manger seriously considers staff suggestions for improving patient safety	74.9 (agreed)
*Whenever pressure builds up, my supervisor/ manager wants us to work faster, even if it means taking shortcuts	46.1 (disagreed)
*My supervisor/ manager overlooks patient safety problems that happen again and again	31.9 (disagreed)
***Frequency of events reported***, overall positive response = 58.1%	
When an error is made, but is noticed and corrected before affecting the patient, how often is this reported?	53.5 (agreed)
When an error is made, but has no potential to harm the patient, how often is this reported?	56.9 (agreed)
When an error is made that could potentially harm the patient but does not, how often is this reported?	63.8 (agreed)
***Overall perceptions of patient safety***, overall positive response = 59.1%	
Patient safety is never sacrificed to get more work done	70.6 (agreed)
Our procedures and systems are good at preventing errors from happening	78.7 (agreed)
*It is just by chance that more serious mistakes don’t happen around here	36.0 (disagreed)
*We have patient safety problems in this unit	51.3 (disagreed)
***Feedback and communication about error***, overall positive response = 61.9%	
We are given feedback about changes put into place based on error reports	55.8 (agreed)
We are informed about medication errors in this unit	62.0 (agreed)
In this unit, we discuss ways to prevent medication errors from happening again	68.0 (agreed)
***Teamwork across units***, overall positive response = 67.7%	
There is good cooperation among hospital units that need to work together	72.9 (agreed)
Hospital units work well together to provide the best care for patients	82.8 (agreed)
*Hospital units do not coordinate well with each other	57.5 (disagreed)
*It is often unpleasant to work with staff from other hospital units	57.5 (disagreed)
***Management support for patient safety***, overall positive response = 75.4%	
Hospital management provides a work environment that promotes patient safety	87.0 (agreed)
The actions of hospital management show that patient safety is a top priority	84.2 (agreed)
Hospital management seems interested in patient safety only after an error happens	54.9 (agreed)
***Teamwork within units***, overall positive response = 82.1%	
People support one another in this unit	81.1 (agreed)
When a lot of work needs to be done quickly, we work as a team to get the work done	83.4 (agreed)
In this unit, people treat each other with respect	81.9 (agreed)
***Organisational learning—continuous improvement***, Overall positive response = 85.8%	
We are actively doing things to improve patient safety	90.2 (agreed)
After we make changes to improve patient safety, we evaluate their effectiveness	81.3 (agreed)

*Reverse scored negatively worded statement

** Calculated from the mean items within each composite

Non-punitive response to errors—all individual items contributing to this HSOPS composite attracted a low level of positive response, this was particularly the case for items relating to staff concerns over errors being kept in their personnel files (26.2%), and the perception that errors counted against them (14.6%). There were highly statistically significant associations with mean composite agreement and gender (females most positive, Χ^2^ (1, N = 1547) = 8.23, p<0.005), age (older most positive, Χ^2^ (4, N = 1555) = 11.62, p<0.05) and experience as a healthcare professional (the most experienced being most positive, Χ^2^ (5, N = 1536) = 18.42, p<0.005).

Staffing–while all responses attracted a low level of positive response, this was particularly the case for work pressures and speed of work (23.5%). There were highly statistically significant associations with mean composite agreement and healthcare professions (doctors most positive and pharmacists least, Χ^2^ (2, N = 1494) = 42.06, p<0.001), age (youngest least and oldest most positive, Χ^2^ (4, N = 1564) = 28.89, p<0.001) and experience as a health professional (positive responses increasing with experience, Χ^2^ (1, N = 1550) = 42.06, p<0.001).

For those ten composites with higher mean agreement, several items had less than half responding positively. There were issues around: supervisors/ managers overlooking recurring patient safety problems (31.9% positive); that it was due to chance that serious errors did not occur (36.0%); problems occurring when exchanging information across hospital units (42.9%); staff being able to ask questions if things did not seem right (44.0%); that at particular pressure points supervisors/ managers wanted staff to work faster, even if this required shortcuts to be taken (46.1%); and staff feeling able to question those in positions of authority (46.6%).

More detailed data on the responses to individual items within each composite are given in S1 File.

### Focus groups

#### Demographics of participants

Two hundred and ninety-five survey respondents (18.4%) expressed interest in participating in focus groups. Nine focus groups were conducted (duration 45–60 minutes), at which point data saturation was deemed to have been achieved. Fifty-four individuals from different disciplines participated, with just under half (n = 26, 48.1%) being nurses, followed by 18 (33.3%) pharmacists and 10 (18.5%) doctors. Most were highly experienced with only 11 (20.4%) having <5 years of experience. During the focus groups, there was wide-ranging discussion across the spectrum of medication errors of prescribing, administration and dispensing.

#### Behavioural determinants associated with errors

Themes and subthemes relating to safety culture identified during focus group discussions are mapped to TDF behavioural determinants, with illustrative quotes provided for each.

**A. Social/professional role and identity** (a coherent set of behaviours and displayed personal qualities of an individual in a social or work setting)

#### 1. Doctors reliance on pharmacists to correct errors

During discussion, it emerged that there were instances where doctors would rely on pharmacists to correct their prescribing errors and this led to complacency around prescribing,

*‘Yes*. *Most of the physicians make a medication error and wait for the pharmacist to correct it*.*’* (Focus Group [FG] 5 Pharmacist 4)

#### 2. Doctors reluctance to alter other doctors’ prescribing

During one focus group, there was concern that doctors were unwilling to alter prescriptions written by other doctors, particularly for doctors from other specialities. The doctors considered this to be the responsibility of the original prescriber, even if a prescribing error had been made and initial prescriber was unavailable,

*‘This will happen when you’re in the Ob-Gyn* [obstetrics and gynaecology] *setup*. *If one physician came from Hamad from other… from cardiac or other site*, *if they write any prescription*, *if you call the Ob-Gyn doctor here*, *the on duty doctor*, *she will never agree to change because she will say it’s an order from the consultant from cardiology or neurology*.*’* (FG7 Pharmacist 4)

#### 3. Lack of recognition of the role of nurses

Some of the nurses described that they were often omitted from discussions around patient care and decision making, even when present on ward rounds or meetings. There were instances where discussions took place in a different language,

*‘Even I’m noting that during the rounds*, *team decisions*, *the nurses are not informed*. *Sometimes they* [the doctors] *are discussing in Arabic*. *The nurse*, *she cannot understand their plan and what is the decision*.*’* (FG3 Nurse 1)

#### 4. Policy non-adherence

Health professionals not adhering to various policies was considered a cause of medication errors,

*‘Not abiding the… complying with the policies’* (FG2 Doctor 2)

*‘There are seven or eight points that the pharmacist should check*. *If the pharmacist*, *for example*, *dispensed the wrong medication it means that he didn’t follow the policy*.*’* (FG5 Pharmacist 4)

**B. Emotions** (a complex reaction pattern, involving experiential, behavioural, and physiological elements, by which the individual attempts to deal with a personally significant matter or event)

#### 1. Stress leading to medication errors

Stress and high pressure situations were described in all focus groups as influences on medication errors. While workload was a common factor leading to stress, patients themselves could also put undue pressure and hence stress of health professionals,

*‘And I think that probably the stresses of the work* [lead to errors].*’* (FG1 Doctor 2)

*‘And parents are too tense than they are… even the parents they are too much angry*. *Yeah*, *they will scold the staff then like that time they will get pressure*.*’* (FG7 Nurse 3)

**C. Environmental Context and Resources** (any circumstance of a person's situation or environment that discourages or encourages the development of skills and abilities, independence, social competence, and adaptive behaviour)

Much of the discussion centred on aspects of environmental context and resources as key influences leading to medication errors. These were discussed by all participants in all focus groups. There were several key themes within this domain.

#### 1. Workload issues leading to medication errors

Workload issues were discussed by doctors, nurses and pharmacists. Doctors believed one of the reasons for errors to happen was the heavy workload that they had.

*‘Too many patients*. *Labour ward is full*, *you know*, *too many patients for the residents to see*.*’* (FG1 Doctor 2)

*‘Yeah*, *I’m working in emergency*. *So what I feel is it’s too much… sometime it is too busy and doctors are giving too much orders…they cannot to cope with the situation*.*’* (FG1 Nurse 1)

One pharmacist noted that the excessive workload for the doctor can lead to errors occurring and that this workload also put pressure on other health professionals which could compound errors.

*‘There are two problems here*, *a load on the physician that can lead to many mistakes and a load on the pharmacist because he needs to dispense medication for this patient and at the same time answer the questions of physician*, *nurses*.*’* (FG5 Pharmacist 4)

One of the nurses also explained that the main cause of errors committed by junior medical staff was workload rather than lack of knowledge.

*‘And this is why the medication errors are also increasing*, *so it’s not always related to the knowledge of the resident*. *And if the resident is overloaded because he has to document for all the patients and see all the patients and he is receiving calls from other units as well’* (FG3 Nurse 3)

#### 2. Lack of staff at key times

Closely related to workload issues was a critical lack of staff at key times such as weekends and evening which could compromise patient safety.

*‘On the whole days of the week*, *there is complete staff*, *complete number of physicians*. *In weekend*, *well*, *only one physician is doing the whole work*.*’* (FG4 Doctor 2)

*‘Especially the areas like emergency*, *less staff*. *They will be get… too tense by the patients and they just want to do the things for faster*. *so it will make errors*. (FG2 Nurse 1)

#### 3. System-related issues

Discussion also centred on key issues related to the systems in operation in various wards and departments. There was particular concern over the implementation of Cerner (electronic health record system for hospitals, health care providers, clinics) from doctors, nurses and pharmacists.

*‘The electronic system is not robust*, *and I mean*, *the hardware is not good enough*.*’* (FG2 Doctor 1)

*‘We have now to concentrate on the mistakes or medication errors happening by the prescribing system*.*’* (FG5 Pharmacist 2)

One senior doctor commented that following implementation of Cerner, fewer checks were being performed compared to the previous paper-based system.

*‘Before it was like*, *when you have the hard copy of medication profile*, *someone is checking and countersigning*. *Now in the system*, *it* [checking] *is not there as far as I know*.*’* (FG1 Doctor 2)

Themes and subthemes for those behavioural determinants less related to safety culture are summarised in Table 5.

**Table 5:** A summary of TDF domains and themes (less related to culture) relating to causes of medication errors.

TDF Domain	Subtheme	Illustrative quotes
**Knowledge**	1. Lack of medication related knowledge	‘So coming to the nursing knowledge regarding the dose. I will never believe they have that much knowledge about the doses…’ (FG1 Doctor 1)
	2. Knowledge is limited to a particular speciality/area	‘If we’re dealing with the general hospital, medicine department they have good orientation regarding medication, but if you go to ortho [orthopaedics] or surgery, really their knowledge about medication is very low.’ (FG5 Pharmacist 3)
	3. Lack of knowledge attributed to staff induction	‘Proper induction, you know, they should have proper induction regarding the medication, the medications that are used, how you do the checking and things like that. Nothing is done.’ (FG1 Doctor 2)
	4. Need for continuing professional development to reduce medication errors	‘There is too much error in this area, they can provide another or a new continuous education for this field. It’s very important and this can prevent such error.’ (FG7 Nurse 1)
**Skills**	1. Suboptimal medication related skills	‘We need to think about the administration. I have seen plenty of times the paper on which they [nurses] have written the calculation and it’s wrong, actually most of the time.’ (FG4 Pharmacist 1)
**Beliefs about Capabilities**	1. Lack of medication related competence	‘But you think it’s… it’s… it’s valid to let the nurses check the dose before administering? No, I don’t think it’s possible. For me, I feel it’s impossible for them to check the correct dose.’ (FG1 Doctor 1)
	2. Overconfidence leading to medication errors	‘Overconfidence with some particular medicines like I have been with this medicine for many years and I know by heart’ (FG1 Pharmacist 2)
**Goals**	1. Promoting patient safety	‘But you know, serious errors are part of the package, you know. As we save lives, we are not ensuring… I mean, we should expect that we cannot have zero even serious errors because we are human beings’. (FG5 Pharmacist 1)

## Discussion

### Key findings

Our study of the causes of medication errors in Qatar highlighted that the key composites of patient safety culture which merit attention are: non-punitive response to errors; staffing; communication openness; handoffs and transitions; and supervisor/manager expectations and actions promoting patient safety. During focus group discussions, specific TDF determinants suggested as being potentially associated with these composites were: social/professional role and identity; emotions; and environmental context and resources. Thematic analysis identified issues of doctors relying on pharmacists to correct their errors and being reluctant to alter the prescribing of fellow doctors. There was a lack of recognition of nurses’ roles and frequent policy non-adherence. Stress was perceived to be a major contributor to errors, as was excessive workload and lack of staff at key times.

### Strengths and weaknesses

The mixed methods design is a key study strength providing quantification of results followed by in-depth explanation. Further strengths are the use of the validated HSOPS tool and embedding psychological behaviour change theory (TDF) within qualitative data generation and analysis [15,19]. There are, however, several limitations hence findings should be interpreted with caution. Self-reported questionnaire responses could not be validated and may have been impacted by response and social desirability biases [22]. While responses were received from healthcare professionals in all HMC hospitals, these may have been skewed towards females and nurses hence there are potential issues of lack of generalisability within Qatar and beyond. Similarly, qualitative findings may not be transferable to other healthcare professionals, settings and countries.

### Interpretation

This mixed methods study has contributed to the expressed need for robustness and rigour in patient safety research within the Middle East [20]. Furthermore, it aligns to the WHO ‘Global Patient Safety Challenge’ calling for action to reduce severe, avoidable medication-related harm by 50% in the next five years [2,3]. Whilst the HSOPS questionnaire has been used within the Middle East [21], this is the first study to publish Qatari data. There are, however, similarities between the Qatari data and those reported by Elmontsri et al. [21], with the lowest agreement scores (and hence of most concern) relating to the composites of non-punitive response to errors; staffing; communication openness; handoffs and transitions; and supervisor/ manager expectations and actions promoting patient safety. Within the two composites of lowest scores (non-punitive response to errors and staffing) there were issues with staff perceiving that errors counted against them and that details of errors committed were kept in their personnel files. This appeared to be an issue for male, younger and less experienced healthcare professionals. Staffing was the other key composite with very low agreement scores, particularly in relation to work pressures and speed of work, with similar statistically significant associations as for the non-punitive response to errors. There may be some merit in initially prioritising any intervention towards these specific groupings.

One limitation of the published studies using the HSOPS is the lack of qualitative research to provide in-depth explanation of the results [21]. The use of behavioural theory within the focus groups in this study identified key determinants which could facilitate intervention development. TDF has been incorporated within intervention developments for smoking cessation, physical activity, hand hygiene, acute low back pain and schizophrenia [28]. To date only one other published study has applied TDF to explore potential causes of medication errors, focusing on prescribing errors in a sample of junior doctors in Scotland [29]. There are some similarities with the findings of this study, most notably within the domains of knowledge and skills, particularly the general lack of medication-related knowledge. While pharmacists can provide support, and indeed doctors were found to rely on pharmacists to correct errors, the HSOPS data and the focus groups identified issues around staff complement and workload, particularly at key times.

TDF domains of social/ professional role and identify, emotions and environmental context and resources are related to organisational safety culture, as defined by ‘Study Group on Human Factors’ [16]. Concerns were expressed around nurses perceiving that their professional role was not recognised leading to poor communication compromising patient safety. This is also reflected in the HSOPS score of ~ 50% agreement for communication openness. There were instances of doctors relying on pharmacists to correct their prescribing errors and, at times, would not alter the prescribing of others, even when errors could potentially lead to patient harm. Themes of environmental context and resources also emerged in the discussions around workload as a leading cause of errors, with lack of staff at key pressure times of evening and weekends. Furthermore, the electronic prescribing and records system was considered to have introduced potential for error. While such systems have been shown to enhance patient safety, others have also highlighted the risky human factors and user-centred design issues that have been encountered [13].

Stress was the main theme which emerged in the TDF emotions domain as a determinant of error, arising due to workload, work pressures and the influence of patients. Issues of workload were also identified in the HSOPS data around staff numbers to handle the workload, working under pressure to do too much, too quickly.

These TDF l determinants which were highlighted as potential contributors to medication errors can be used during the development of behaviour change interventions, defined as ‘coordinated sets of activities designed to change specified behaviour patterns’. These are often complex, consisting of interacting components known as ‘behaviour change techniques’ (BCTs), ‘observable and replicable components designed to change behaviour’ [30]. Michie et al. developed a cross-disciplinary taxonomy of evidence based BCTs [31], mapped to specific TDF domains [32]. Whilst knowledge and skills can be impacted through education and training [31,32], altering aspects of social/ professional role and identity and environmental context and resources are more complex. Indeed, the work of Michie et al. [31,32] did not identify any evidence-based BCTs which mapped reliably to social/professional role and identity. Those for environmental context and resources relating mainly to restructuring the physical environment and providing prompts and cues for safer practice, which in this case would focus on the electronic medication systems [31,32]. Rather than focusing on changing behaviour at the individual practitioner level, action may be required at the organisational strategic level to review policies, structures (including resource allocation and distribution) and processes which aim to promote patient safety culture and minimise harm. Qualitative research focusing on understanding the perspectives of key strategic decision-makers in relation to promoting all aspects of medication safety is warranted.

### Conclusion

This mixed methods study has provided further confirmation of key areas of concern relating to patient safety culture in Qatar. Non-punitive response to errors and staffing had the lowest levels of agreement, followed by communication openness, handoffs and transitions, and supervisor/manager expectations and actions. The qualitative component provided further detail of specific TDF determinants highlighting issues of social/professional role and identity, emotions, and environmental context and resources. Further attention on these issues at strategic and policy levels is required.

## Supporting information

S1 File Responses to each of the HSOPS composites. (DOCX)

S2 File Study questionnaire. (DOCX)
