# Supplementary material for: Perspectives of healthcare professionals in Qatar on causes of medication errors: A mixed methods study of safety culture
Source: PLoS One. 2018 Sep 28;13(9):e0204801. doi: 10.1371/journal.pone.0204801 (PMC6161876; doi:10.1371/journal.pone.0204801)
Supplement: S1 File — (DOCX) [file pone.0204801.s001.docx]

**A MIXED METHODS STUDY OF PATIENT SAFETY CULTURE AND CAUSES OF MEDICATION ERRORS**

**SUPPLEMENTARY FILE**

Table 1. Teamwork within units, responses to HSOPS items (N=1604)

| **Statements** | **Strongly Agree**  **% (n)** | **Agree**  **% (n)** | **Unsure**  **% (n)** | **Disagree**  **% (n)** | **Strongly**  **Disagree**  **% (n)** | **Missing**  **% (n)** | **% positive response (100% the highest positive response to each statement)** |
| --- | --- | --- | --- | --- | --- | --- | --- |
| People support one another in this unit | 23.6 (379) | 57.5 (922) | 10.1 (162) | 4.8  (77) | 2.2  (36) | 1.7  (28) | 81.1 |
| When a lot of work needs to be done quickly, we work as a team to get the work done | 23.1 (370) | 60.3 (968) | 8.6  (138) | 5.0  (80) | 1.2  (20) | 1.7  (28) | 83.4 |
| In this unit, people treat each other with respect | 22.7 (364) | 59.2 (950) | 10.0 (160) | 4.5  (72) | 1.7  (27) | 1.9  (31) | 81.9 |
| Overall positive response = 82.1% | | | | | | | |

Table 2. Supervisor/manager expectations and actions promoting patient safety, responses to HSOPS items (N=1604)

| **Statements** | **Strongly Agree**  **% (n)** | **Agree**  **% (n)** | **Unsure**  **% (n)** | **Disagree**  **% (n)** | **Strongly**  **Disagree**  **% (n)** | **Missing**  **% (n)** | **% positive response (100% the highest positive response to each statement)** |
| --- | --- | --- | --- | --- | --- | --- | --- |
| My supervisor/ manager says a good word when he/she sees a job done according to established patient safety procedures | 21.4 (344) | 51.6 (827) | 13.5 (216) | 8.5  (136) | 3.4  (54) | 1.7  (27) | 73.0 |
| My supervisor/ manger seriously considers staff suggestions for improving patient safety | 20.7 (332) | 54.2 (870) | 14.0 (224) | 6.4  (102) | 2.6  (42) | 2.1  (34) | 74.9 |
| *Whenever pressure builds up, my supervisor/ manager wants us to work faster, even if it means taking shortcuts | 6.3  (101) | 22.9 (367) | 22.3 (357) | 37.5  (601) | 8.6  (138) | 2.5  (40) | 46.1 |
| *My supervisor/ manager overlooks patient safety problems that happen again and again | 11.7 (188) | 39.8 (638) | 13.7 (219) | 22.6  (363) | 9.3  (149) | 2.9  (47) | 31.9 |
| Overall positive response = 56.5% | | | | | | | |

*negatively worded so reverse scored

Table 3. Organisational learning - continuous improvement, responses to HSOPS items (N=1604)

| **Statements** | **Strongly Agree**  **% (n)** | **Agree**  **% (n)** | **Unsure**  **% (n)** | **Disagree**  **% (n)** | **Strongly**  **Disagree**  **% (n)** | **Missing**  **% (n)** | **% positive response (100% the highest positive response to each statement)** |
| --- | --- | --- | --- | --- | --- | --- | --- |
| We are actively doing things to improve patient safety | 33.9 (543) | 56.3 (903) | 5.2  (83) | 1.9  (31) | 0.7  (11) | 2.1  (33) | 90.2 |
| After we make changes to improve patient safety, we evaluate their effectiveness | 20.0 (320) | 61.3 (984) | 10.8 (173) | 4.4  (70) | 1.4  (22) | 2.2  (35) | 81.3 |
| Overall positive response = 85.8% | | | | | | | |

Table 4. Management support for patient safety, responses to HSOPS items (N=1604)

| **Statements** | **Strongly Agree**  **% (n)** | **Agree**  **% (n)** | **Unsure**  **% (n)** | **Disagree**  **% (n)** | **Strongly**  **Disagree**  **% (n)** | **Missing**  **% (n)** | **% positive response (100% the highest positive response to each statement)** |
| --- | --- | --- | --- | --- | --- | --- | --- |
| Hospital management provides a work environment that promotes patient safety | 30.9 (495) | 56.1 (900) | 7.2  (115) | 2.4  (38) | 0.9  (15) | 2.6  (41) | 87.0 |
| The actions of hospital management show that patient safety is a top priority | 35.1 (563) | 49.1 (787) | 8.4  (135) | 2.5  (40) | 1.2  (20) | 3.7  (59) | 84.2 |
| Hospital management seems interested in patient safety only after an error happens | 7.2  (115) | 20.9 (335) | 13.7 (220) | 41.4  (664) | 13.5  (217) | 3.3  (53) | 54.9 |
| Overall positive response = 75.4% | | | | | | | |

Table 5. Overall perceptions of patient safety, responses to HSOPS items (N=1604)

| **Statements** | **Strongly Agree**  **% (n)** | **Agree**  **% (n)** | **Unsure**  **% (n)** | **Disagree**  **% (n)** | **Strongly**  **Disagree**  **% (n)** | **Missing**  **% (n)** | **% positive response (100% the highest positive response to each statement)** |
| --- | --- | --- | --- | --- | --- | --- | --- |
| Patient safety is never sacrificed to get more work done | 23.1 (370) | 47.5 (762) | 11.9 (191) | 11.1  (178) | 3.1  (50) | 3.3  (53) | 70.6 |
| Our procedures and systems are good at preventing errors from happening | 19.6 (314) | 59.1 (948) | 12.1 (194) | 4.4  (70) | 2.4  (39) | 2.4  (39) | 78.7 |
| *It is just by chance that more serious mistakes don’t happen around here | 7.5  (120) | 36.2 (581) | 17.5 (281) | 29.5  (473) | 6.5  (105) | 2.7  (44) | 36.0 |
| *We have patient safety problems in this unit | 7.5  (120) | 20.7 (332) | 17.9 (287) | 39.3  (630) | 12.0  (193) | 2.6  (42) | 51.3 |
| Overall positive response = 59.1% | | | | | | | |

*negatively worded so reverse scored

Table 6. Feedback and communication about error, responses to HSOPS items (N=1604)

| **Statements** | **Always**  **% (n)** | **Most of the time**  **% (n)** | **Some**  **times**  **% (n)** | **Rarely**  **% (n)** | **Never**  **% (n)** | **Missing**  **% (n)** | **% positive response (100% the highest positive response to each statement)** |
| --- | --- | --- | --- | --- | --- | --- | --- |
| We are given feedback about changes put into place based on error reports | 17.3 (278) | 38.5  (618) | 28.7 (461) | 9.5 (153) | 2.7 (43) | 3.2  (51) | 55.8 |
| We are informed about medication errors in this unit | 27.5 (441) | 35.0  (562) | 20.1 (323) | 10.4 (167) | 3.9 (62) | 3.1  (49) | 62.0 |
| In this unit, we discuss ways to prevent medication errors from happening again | 31.7 (509) | 36.3  (582) | 19.3 (310) | 6.7 (108) | 2.3 (37) | 3.6  (58) | 68.0 |
| Overall positive response = 61.9% | | | | | | | |

Table 7. Communication openness, responses to HSOPS items (N=1604)

| **Statements** | **Always**  **% (n)** | **Most of the time**  **% (n)** | **Some**  **times**  **% (n)** | **Rarely**  **% (n)** | **Never**  **% (n)** | **Missing**  **% (n)** | **% positive response (100% the highest positive response to each statement)** |
| --- | --- | --- | --- | --- | --- | --- | --- |
| Staff will speak up freely if they see something that may negatively affect patient care | 21.6 (347) | 39.3  (631) | 25.6 (410) | 8.2 (132) | 2.6 (42) | 2.6  (42) | 60.9 |
| Staff feel free to question the decisions or actions of those with more authority | 14.7 (236) | 31.9  (511) | 28.9 (463) | 15.5 (248) | 6.0 (96) | 3.1  (50) | 46.6 |
| *In this unit, staff are afraid to ask questions when something does not seem right | 7.4  (118) | 17.5  (280) | 28.1 (450) | 25.1 (402) | 18.9 (303) | 3.2  (51) | 44.0 |
| Overall positive response = 50.5% | | | | | | | |

*negatively worded so reverse scored

Table 8. Frequency of events reported, responses to HSOPS items (N=1604)

| **Statements** | **Always**  **% (n)** | **Most of the time**  **% (n)** | **Some**  **times**  **% (n)** | **Rarely**  **% (n)** | **Never**  **% (n)** | **Missing**  **% (n)** | **% positive response (100% the highest positive response to each statement)** |
| --- | --- | --- | --- | --- | --- | --- | --- |
| When an error is made, but is noticed and corrected before affecting the patient, how often is this reported? | 22.8 (366) | 30.7  (493) | 22.8 (366) | 15.1 (243) | 5.1 (82) | 3.4  (54) | 53.5 |
| When an error is made, but has no potential to harm the patient, how often is this reported? | 25.6 (411) | 31.3  (502) | 20.9 (336) | 12.2 (196) | 5.4 (87) | 4.5  (72) | 56.9 |
| When an error is made that could potentially harm the patient but does not, how often is this reported? | 31.9 (512) | 31.9  (511) | 16.7 (268) | 9.3 (149) | 5.0 (81) | 5.2  (83) | 63.8 |
| Overall positive response = 58.1% | | | | | | | |

Table 9. Teamwork across units, responses to HSOPS items (N=1604)

| **Statements** | **Strongly Agree**  **% (n)** | **Agree**  **% (n)** | **Unsure**  **% (n)** | **Disagree**  **% (n)** | **Strongly**  **Disagree**  **% (n)** | **Missing**  **% (n)** | **% positive response (100% the highest positive response to each statement)** |
| --- | --- | --- | --- | --- | --- | --- | --- |
| There is good cooperation among hospital units that need to work together | 15.7 (252) | 57.2 (918) | 15.4 (247) | 6.5  (104) | 1.9  (30) | 3.3  (53) | 72.9 |
| Hospital units work well together to provide the best care for patients | 31.7 (509) | 51.1 (820) | 9.4  (151) | 2.7  (44) | 1.2  (19) | 3.8  (61) | 82.8 |
| *Hospital units do not coordinate well with each other | 5.4  (86) | 18.1 (291) | 15.5 (249) | 45.6  (732) | 11.9  (191) | 3.4  (55) | 57.5 |
| *It is often unpleasant to work with staff from other hospital units | 3.2  (51) | 16.6 (266) | 19.5 (313) | 46.4  (745) | 11.1  (178) | 3.2  (51) | 57.5 |
| Overall positive response = 67.7% | | | | | | | |

*negatively worded so reverse scored

Table 10. Staffing, responses to HSOPS items (N=1604)

| **Statements** | **Strongly Agree**  **% (n)** | **Agree**  **% (n)** | **Unsure**  **% (n)** | **Disagree**  **% (n)** | **Strongly**  **Disagree**  **% (n)** | **Missing**  **% (n)** | **% positive response (100% the highest positive response to each statement)** |
| --- | --- | --- | --- | --- | --- | --- | --- |
| We have enough staff to handle the workload | 13.2 (211) | 41.5 (665) | 14.7 (235) | 21.8  (349) | 7.5  (120) | 1.5  (24) | 54.7 |
| *We use more locum staff than is best for patient care | 9.0  (145) | 29.5 (473) | 26.2 (421) | 22.4  (360) | 8.1  (130) | 4.7  (75) | 30.5 |
| *We work under pressure trying to do too much, too quickly | 17.5 (280) | 40.0 (641) | 17.2 (276) | 20.2  (324) | 3.3  (53) | 1.9  (30) | 23.5 |
| Overall positive response = 36.2% | | | | | | | |

*negatively worded so reverse scored

Chi-square analysis

1. Association between age and positive responses

|  | 20-29 years | 30-39 years | 40-49 years | 50-59 years | 60-69 years |
| --- | --- | --- | --- | --- | --- |
| Positive responses | 359 | 733 | 411 | 140 | 20 |
| Negative responses | 790 | 1220 | 603 | 305 | 54 |

Three items hence each individual represented by three counts

Youngest least and oldest most positive, Χ^2^ (4, N=1564)=28.89, p<0.001.

2. Association between years of experience and positive responses

|  | <1 year | 1-5 years | 5-10 years | 11-15 years | 16-20 years | >20 years |
| --- | --- | --- | --- | --- | --- | --- |
| Positive responses | 16 | 292 | 459 | 416 | 240 | 292 |
| Negative responses | 50 | 601 | 911 | 596 | 329 | 391 |

Three items hence each individual represented by three counts

Positive responses increase with experience, Χ^2^ (1, N=1550)=42.06, p<0.001.

3. Association between profession and positive responses

|  | Doctors | Nurses | Pharmacists |
| --- | --- | --- | --- |
| Positive responses | 258 | 1236 | 169 |
| Negative responses | 366 | 1966 | 433 |

Three items hence each individual represented by three counts

Doctors most positive and pharmacists least, Χ^2^ (2, N=1494)=42.06, p<0.001.Table 11. Handoffs and transitions, responses to HSOPS items (N=1604)

| **Statements** | **Strongly Agree**  **% (n)** | **Agree**  **% (n)** | **Unsure**  **% (n)** | **Disagree**  **% (n)** | **Strongly**  **Disagree**  **% (n)** | **Missing**  **% (n)** | **% positive response (100% the highest positive response to each statement)** |
| --- | --- | --- | --- | --- | --- | --- | --- |
| *Things get missed when transferring patients from one unit to another | 5.3  (85) | 19.6 (314) | 18.2 (292) | 43.0  (690) | 10.7  (172) | 3.2  (51) | 53.7 |
| *Important patient care information is often lost during shift changes | 4.2  (68) | 17.3 (277) | 14.2 (227) | 45.8  (735) | 15.0  (240) | 3.6  (57) | 60.8 |
| *Problems often occur in the exchange of information across hospital units | 4.6  (73) | 26.6 (426) | 22.4 (359) | 36.0  (578) | 6.9  (111) | 3.6  (57) | 42.9 |
| *Shift changes are problematic for patients in this hospital | 5.4  (86) | 17.3 (278) | 18.2 (292) | 40.6  (652) | 14.5  (232) | 4.0  (64) | 55.1 |
| Overall positive response = 53.1% | | | | | | | |

*negatively worded so reverse scored

Table 12. Non-punitive response to errors, responses to HSOPS items (N=1604)

| **Statements** | **Strongly Agree**  **% (n)** | **Agree**  **% (n)** | **Unsure**  **% (n)** | **Disagree**  **% (n)** | **Strongly**  **Disagree**  **% (n)** | **Missing**  **% (n)** | **% positive response (100% the highest positive response to each statement)** |
| --- | --- | --- | --- | --- | --- | --- | --- |
| *Staff feel like errors count against them | 12.1 (194) | 37.0 (593) | 22.6 (363) | 22.9  (368) | 3.3  (53) | 2.1  (33) | 26.2 |
| *When an error is reported, it feels like the person is being reported, not the problem | 11.5 (185) | 38.1 (611) | 17.3 (278) | 25.7  (413) | 5.4  (86) | 1.9  (31) | 31.1 |
| *Staff worry that errors they make are kept in their personnel file | 18.2 (292) | 51.2 (821) | 13.5 (216) | 12.0  (193) | 2.6  (42) | 2.5  (40) | 14.6 |
| Overall positive response = 24.0% | | | | | | | |

*negatively worded so reverse scored

Chi-square analysis

1. Association between age and positive responses

|  | 20-29 years | 30-39 years | 40-49 years | 50-59 years | 60-69 years |
| --- | --- | --- | --- | --- | --- |
| Positive responses | 276 | 447 | 273 | 126 | 24 |
| Negative responses | 882 | 1518 | 738 | 330 | 51 |

Three items hence each individual represented by three counts

Older most positive, Χ^2^ (4, N=1555)=11.62, p<0.05.

2. Association between years of experience and positive responses

|  | <1 year | 1-5 years | 5-10 years | 11-15 years | 16-20 years | >20 years |
| --- | --- | --- | --- | --- | --- | --- |
| Positive responses | 13 | 201 | 299 | 269 | 146 | 201 |
| Negative responses | 46 | 688 | 1084 | 742 | 423 | 493 |

Three items hence each individual represented by three counts

Most experienced being most positive, Χ^2^ (5, N=1536)= 18.42, p<0.005.

3. Association between gender and positive responses

|  | Female | Male |
| --- | --- | --- |
| Positive responses | 855 | 279 |
| Negative responses | 2490 | 1017 |

Three items hence each individual represented by three counts

Females most positive, Χ^2^ (1, N=1547)=8.23, p<0.005.
