# Supplementary material for: Perspectives of healthcare professionals in Qatar on causes of medication errors: A mixed methods study of safety culture
Source: PLoS One. 2018 Sep 28;13(9):e0204801. doi: 10.1371/journal.pone.0204801 (PMC6161876; doi:10.1371/journal.pone.0204801)
Supplement: S2 File — (DOCX) [file pone.0204801.s002.docx]

**We will start with some information about you which will help with the analysis of the data.**

**SECTION 1: Demographics**

**1. Are you:**

| 🞎 a. male | 🞎 b. female |
| --- | --- |

**2. Your age in years is:**

| 🞎 a. under 20 | 🞎 c. 30 - 39 | 🞎 e. 50 - 59 |
| --- | --- | --- |
| 🞎 b. 20 - 29 | 🞎 d. 40 - 49 | 🞎 f. 60 - 69 |
|  |  | 🞎 g. over 69 |

**3. Your country of origin is:**

| 🞎 a. Qatar | 🞎 d. India | 🞎 h. Philippines |
| --- | --- | --- |
| 🞎 b. other GCC | 🞎 e. Jordan | 🞎 i. Sudan |
| 🞎 c. Egypt | 🞎 f. Lebanon | 🞎 j. UK |
|  | 🞎 g. Palestine | 🞎 k. Other, please specify: |
|  |  |  |

**4. Your country of receiving entry-to-practice degree was:**

| 🞎 a. Qatar | 🞎 d. India | 🞎 h. Philippines |
| --- | --- | --- |
| 🞎 b. other GCC | 🞎 e. Jordan | 🞎 i. Sudan |
| 🞎 c. Egypt | 🞎 f. Lebanon | 🞎 j. UK |
|  | 🞎 g. Palestine | 🞎 k. Other, please specify: |
|  |  |  |

**5. Your country of receiving highest academic degree was:**

| 🞎 a. Qatar | 🞎 d. India | 🞎 h. Philippines |
| --- | --- | --- |
| 🞎 b. other GCC | 🞎 e. Jordan | 🞎 i. Sudan |
| 🞎 c. Egypt | 🞎 f. Lebanon | 🞎 j. UK |
|  | 🞎 g. Palestine | 🞎 k. Other, please specify: |
|  |  |  |

**6. Your highest academic degree is:**

| 🞎 a. BSc (Nursing) | 🞎 e. MSc | 🞎 i. PhD |
| --- | --- | --- |
| 🞎 b. BSc (Pharm) | 🞎 f. MBBS or MBChB | 🞎 j. Additional professional qualification, please specify: |
| 🞎 c. MPharm | 🞎 g. MD |  |
| 🞎 d. PharmD | 🞎 h. HND/Diploma |  |

**7. How long have you worked in this hospital?**

| 🞎 a. < 1 year | 🞎 c. 6 to 10 years | 🞎 e. 16 to 20 years |
| --- | --- | --- |
| 🞎 b. 1 to 5 years | 🞎 d. 11 to 15 years | 🞎 f. 21 years or more |

**8. How long have you worked in your current hospital work area/unit?**

| 🞎 a. < 1 year | 🞎 c. 6 to 10 years | 🞎 e. 16 to 20 years |
| --- | --- | --- |
| 🞎b. 1 to 5 years | 🞎 d. 11 to 15 years | 🞎 f. 21 years or more |

**9. Typically, how many hours per week do you work in this hospital?**

| 🞎a. < 20 hours per week | 🞎c. 40 to 59 hours per week |  |
| --- | --- | --- |
| 🞎 b. 20 to 39 hours per week | 🞎d. > 60 hours per week |  |

**10. What is your role in this hospital? Select ONE answer that best describes your role**

| 🞎 a. Consultant Physician | 🞎 d. Clinical Nurse Educator | 🞎 g. Clinical Pharmacy Specialist |
| --- | --- | --- |
| 🞎 b. Specialist Physician | 🞎 e. Specialist Nurse | 🞎 h. Clinical Pharmacist |
| 🞎 c. Resident Physician | 🞎 f. Nurse | 🞎 i. Pharmacist |
|  |  | 🞎 j. Other, please specify: |
|  |  |  |

**11. In your role, do you typically have direct interaction or contact with patients?**

| 🞎 a. YES, I typically have direct interaction or contact with patients | 🞎 b. NO, I typically do NOT have direct interaction or contact with patients |
| --- | --- |

**12. What are your primary roles in the medicines process? (Tick all that apply)**

| 🞎a. prescribing | 🞎 c. administering | 🞎 e. Other, please specify: |
| --- | --- | --- |
| 🞎 b. preparation & dispensing | 🞎 d. monitoring |  |

**13. How long have you worked in your current role?**

| 🞎a. < 1 year | 🞎 c. 6 to 10 years | 🞎 e. 16 to 20 years |
| --- | --- | --- |
| 🞎 b. 1 to 5 years | 🞎 d. 11 to 15 years | 🞎 f. 21 years or more |

**14. How long have you practiced your profession in Qatar?**

| 🞎a. < 1 year | 🞎 c. 6 to 10 years | 🞎 e. 16 to 20 years |
| --- | --- | --- |
| 🞎 b. 1 to 5 years | 🞎 d. 11 to 15 years | 🞎 f. 21 years or more |

**Thank you. The next section is about your attitude and beliefs about medication errors.**

**SECTION 2:**

| **Hospital Survey on Patient Safety**  **Reprinted/translated with permission from the Agency for Healthcare Research and Quality (an Agency of the United States Department of Health and Human Services); Rockville, Maryland USA**  Internet Citation: International Use of the Surveys on Patient Safety Culture. December 2014. Agency for Healthcare Research and Quality, Rockville, MD. http://www.ahrq.gov/professionals/quality-patient-safety/patientsafetyculture/pscintusers.html |
| --- |
| **Please allow 30 minutes to complete this survey. If you do not wish to answer a question, or if a question does not apply to you, you may leave your answer blank.** |

**SECTION A: Your Work Area/Unit**

**In this survey, think of your “unit” as the work area, department, or clinical area of the hospital where you spend *most* of your work time or provide *most* of your clinical services.**

**What is your primary work area or unit in this hospital? Select ONE answer.**

| 🞏 | a. More than one hospital unit/No specific unit | | |  |  |  |
| --- | --- | --- | --- | --- | --- | --- |
| 🞏 | b. Medicine (non-surgical) | 🞏 | g. Intensive care unit (any type) | | 🞏 | l. Radiology |
| 🞏 | c. Surgery | 🞏 | h. Psychiatry/mental health | | 🞏 | m. Anesthesiology |
| 🞏 | d. Obstetrics & Gynecology | 🞏 | i. Rehabilitation | | 🞏 | n. Other, please specify: |
| 🞏 | e. Pediatrics | 🞏 | j. Pharmacy services | |  |  |
| 🞏 | f. Emergency department | 🞏 | k. Laboratory | |  |  |

**Please indicate your agreement or disagreement with the following statements about your work area/unit**

| **Think about your hospital work area/unit…** | **Strongly Disagree** ⯆ | **Disagree** ⯆ | **Neither** ⯆ | **Agree** ⯆ | **Strongly Agree** ⯆ |
| --- | --- | --- | --- | --- | --- |
| 1. People support one another in this unit | 🞎1 | 🞎2 | 🞏3 | 🞎4 | 🞏5 |
| 2. We have enough staff to handle the workload | 🞎1 | 🞎2 | 🞏3 | 🞎4 | 🞏5 |
| 3. When a lot of work needs to be done quickly, we work together as a team to get the work done | 🞎1 | 🞎2 | 🞏3 | 🞎4 | 🞏5 |
| 4. In this unit, people treat each other with respect | 🞎1 | 🞎2 | 🞏3 | 🞎4 | 🞏5 |
| 5. Staff in this unit work longer hours than is best for patient care | 🞎1 | 🞎2 | 🞏3 | 🞎4 | 🞏5 |
| 6. We are actively doing things to improve patient safety | 🞎1 | 🞎2 | 🞏3 | 🞎4 | 🞏5 |
| 7. We use more agency/ temporary staff than is best for patient care | 🞎1 | 🞎2 | 🞏3 | 🞎4 | 🞏5 |
| 8. Staff feel like their errors count against them | 🞎1 | 🞎2 | 🞏3 | 🞎4 | 🞏5 |
| 9. Errors have led to remedial action and positive changes here | 🞎1 | 🞎2 | 🞏3 | 🞎4 | 🞏5 |

**SECTION A: Your Work Area/Unit (continued)**

| **Think about your hospital work area/unit…** | **Strongly Disagree** ⯆ | **Disagree** ⯆ | **Neither** ⯆ | **Agree** ⯆ | **Strongly Agree** ⯆ |
| --- | --- | --- | --- | --- | --- |
| 10. It is just by chance that more serious mistakes don’t happen around here | 🞎1 | 🞎2 | 🞏3 | 🞎4 | 🞏5 |
| 11. When one area in this unit gets really busy, others help out | 🞎1 | 🞎2 | 🞏3 | 🞎4 | 🞏5 |
| 12. When an error is reported, it feels like the person is being reported, not the problem | 🞎1 | 🞎2 | 🞏3 | 🞎4 | 🞏5 |
| 13. After we make changes to improve patient safety, we evaluate their effectiveness | 🞎1 | 🞎2 | 🞏3 | 🞎4 | 🞏5 |
| 14. We work under pressure trying to do too much, too quickly | 🞎1 | 🞎2 | 🞏3 | 🞎4 | 🞏5 |
| 15. Patient safety is never sacrificed to get more work done | 🞎1 | 🞎2 | 🞏3 | 🞎4 | 🞏5 |
| 16. Staff worry that errors they make are kept in their personnel file | 🞎1 | 🞎2 | 🞏3 | 🞎4 | 🞏5 |
| 17. We have patient safety problems in this unit | 🞎1 | 🞎2 | 🞏3 | 🞎4 | 🞏5 |
| 18. Our procedures and systems are good at preventing errors from happening | 🞎1 | 🞎2 | 🞏3 | 🞎4 | 🞏5 |

**SECTION B: Your Supervisor/Manager**

**Please indicate your agreement or disagreement with the following statements about your immediate supervisor/manager or person to whom you directly report.**

|  | **Strongly Disagree** ⯆ | **Disagree** ⯆ | **Neither** ⯆ | **Agree** ⯆ | **Strongly Agree** ⯆ |
| --- | --- | --- | --- | --- | --- |
| 1. My supervisor/manager says a good word when he/she sees a job done according to established patient safety procedures | 🞎1 | 🞎2 | 🞏3 | 🞎4 | 🞏5 |
| 2. My supervisor/manager seriously considers staff suggestions for improving patient safety | 🞎1 | 🞎2 | 🞏3 | 🞎4 | 🞏5 |
| 3. Whenever pressure builds up, my supervisor/manager wants us to work faster, even if it means taking shortcuts | 🞎1 | 🞎2 | 🞏3 | 🞎4 | 🞏5 |
| 4. My supervisor/manager overlooks patient safety problems that happen again and again | 🞎1 | 🞎2 | 🞏3 | 🞎4 | 🞏5 |

**SECTION C: Communications**

**How often do the following things happen in your work area/unit?**

| **Think about your hospital work area/unit…** | **Never** ⯆ | **Rarely** ⯆ | **Some-times** ⯆ | **Most of the time** ⯆ | **Always** ⯆ |
| --- | --- | --- | --- | --- | --- |
| 1. We are given feedback about changes put into place based on error reports | 🞎1 | 🞎2 | 🞏3 | 🞎4 | 🞏5 |
| 2. Staff will speak up freely if they see something that may negatively affect patient care | 🞎1 | 🞎2 | 🞏3 | 🞎4 | 🞏5 |
| 3. We are informed about errors in this unit | 🞎1 | 🞎2 | 🞏3 | 🞎4 | 🞏5 |
| 4. Staff feel free to question the decisions or actions of those with more authority | 🞎1 | 🞎2 | 🞏3 | 🞎4 | 🞏5 |
| **SECTION C: Communications (continued)** | **Never** ⯆ | **Rarely** ⯆ | **Some-times** ⯆ | **Most of the time** ⯆ | **Always** ⯆ |
| 5. In this unit, we discuss ways to prevent errors from happening again | 🞎1 | 🞎2 | 🞏3 | 🞎4 | 🞏5 |
| 6. Staff are afraid to ask questions when something does not seem right | 🞎1 | 🞎2 | 🞏3 | 🞎4 | 🞏5 |

**SECTION D: Frequency of Errors Reported**

**In your hospital work area/unit, when the following errors happen, *how often are they reported?***

|  | **Never** ⯆ | **Rarely** ⯆ | **Some-times** ⯆ | **Most of the time** ⯆ | **Always** ⯆ |
| --- | --- | --- | --- | --- | --- |
| 1. When an error is made, but is *noticed and corrected before affecting the patient*, how often is this reported? | 🞎1 | 🞎2 | 🞏3 | 🞎4 | 🞏5 |
| 2. When an error is made, but has *no potential to harm the patient*, how often is this reported? | 🞎1 | 🞎2 | 🞏3 | 🞎4 | 🞏5 |
| 3. When an error is made that *could potentially harm the patient* but does not, how often is this reported? | 🞎1 | 🞎2 | 🞏3 | 🞎4 | 🞏5 |

**SECTION E: Patient Safety Grade**

**Please give your work area/unit in this hospital an overall grade on patient safety.**

| 🞎 | 🞎 | 🞎 | 🞎 | 🞎 |
| --- | --- | --- | --- | --- |
| **A**  Excellent | **B**  Very Good | **C**  Acceptable | **D**  Poor | **E**  Failing |

**SECTION F: Your Hospital**

**Please indicate your agreement or disagreement with the following statements about your hospital.**

| **Think about your hospital…** | **Strongly Disagree** ⯆ | **Disagree** ⯆ | **Neither** ⯆ | **Agree** ⯆ | **Strongly Agree** ⯆ |
| --- | --- | --- | --- | --- | --- |
| 1. Hospital management provides a work environment that promotes patient safety | 🞎1 | 🞎2 | 🞏3 | 🞎4 | 🞏5 |
| 2. Hospital units do not coordinate well with each other | 🞎1 | 🞎2 | 🞏3 | 🞎4 | 🞏5 |
| 3. Things get missed when transferring patients from one unit to another | 🞎1 | 🞎2 | 🞏3 | 🞎4 | 🞏5 |
| 4. There is good cooperation among hospital units that need to work together | 🞎1 | 🞎2 | 🞏3 | 🞎4 | 🞏5 |
| 5. Important patient care information is often lost during shift changes | 🞎1 | 🞎2 | 🞏3 | 🞎4 | 🞏5 |
| 6. It is often unpleasant to work with staff from other hospital units | 🞎1 | 🞎2 | 🞏3 | 🞎4 | 🞏5 |
| 7. Problems often occur in the exchange of information across hospital units | 🞎1 | 🞎2 | 🞏3 | 🞎4 | 🞏5 |
| 8. The actions of hospital management show that patient safety is a top priority | 🞎1 | 🞎2 | 🞏3 | 🞎4 | 🞏5 |

| **SECTION F: Your Hospital (continued)** | **Strongly Disagree** ⯆ | **Disagree** ⯆ | **Neither** ⯆ | **Agree** ⯆ | **Strongly Agree** ⯆ |
| --- | --- | --- | --- | --- | --- |
| 9. Hospital management seems interested in patient safety only after an error happens | 🞎1 | 🞎2 | 🞏3 | 🞎4 | 🞏5 |
| 10. Hospital units work well together to provide the best care for patients | 🞎1 | 🞎2 | 🞏3 | 🞎4 | 🞏5 |
| 11. Shift changes are problematic for patients in this hospital | 🞎1 | 🞎2 | 🞏3 | 🞎4 | 🞏5 |
